# The effect of resistance training on physical function is associated with changes in serum albumin redox state in middle-aged and older Japanese adults: a Quasi-experimental study

**DOI:** 10.3389/fphys.2025.1649300

**Published:** 2025-09-17

**Authors:** Takuya Shibasaki, Hirohiko Nakamura, Yuka Kurosaka, Shuji Sawada, Kazuhiro Miyaji, Shuichi Machida

**Affiliations:** ^1^ Health Care & Nutritional Science Institute, Morinaga Milk Industry Co., Ltd., Zama, Japan; ^2^ Faculty of Health and Sports Science, Juntendo University, Inzai, Japan; ^3^ Graduate School of Health and Sports Science, Juntendo University, Inzai, Japan

**Keywords:** albumin, exercise, frailty, protein, sarcopenia, biomarker, mercaptoalbumin, nutrition

## Abstract

**Background:**

Resistance training is important for improving physical function in middle-aged and older adults. The fraction of mercaptoalbumin in total serum albumin, represented as f(HMA), is an indicator of physical function and protein nutritional status in humans. However, it is unclear whether the effects of resistance training on physical function are associated with changes in f(HMA). This study was aimed at examining the relationship between f(HMA) and the effects of resistance training in healthy middle-aged and older Japanese adults.

**Methods:**

The study included 43 healthy community dwelling middle-aged and elderly individuals (10 males and 33 females, aged 67.3 ± 8.0 years). They were engaged in a low-load, body-weight-based resistance training program using an elastic band twice a week for 12 weeks under supervision. Anthropometric data, 6-meter gait speed, blood biochemistry, and dietary macronutrient intake were collected before and after the training intervention. The relationships between serum nutritional parameters and gait speed or their rate of change were examined using multivariate linear regression analysis.

**Results:**

Before intervention, f(HMA) showed a significant positive correlation with the usual (*β* = 0.326, *P* = 0.045) and maximum (*β* = 0.331, *P* = 0.036) gait speeds. The changing rate of maximal gait speed showed a significant positive correlation with the rate of increase in f(HMA) (*β* = 0.456, *P* = 0.004).

**Conclusion:**

Serum f(HMA) increased with improvements in physical function through resistance training in middle-aged and older adults.

## 1 Introduction

Decreased physical function is a serious health concern in elderly individuals, with slow gait speed being a leading cause of decreased activities of daily living (ADLs). Furthermore, it increases the risk of falls (Colón-Emeric et al., 2024), disability (Perera et al., 2016), cognitive impairment (Peng et al., 2020; Taniguchi et al., 2017), and even mortality (Abe et al., 2019; Toots et al., 2013). Resistance training is a useful strategy for preservation and improvement of physical function (Yoshimura et al., 2017). It not only increases skeletal muscle mass but also improves gait speed in middle-aged and older adults (Mende et al., 2022).

Sufficient intake of nutrients, especially dietary proteins, is necessary to maximize the effects of training (Deutz et al., 2014). Protein supplementation could overcome anabolic resistance by activating metabolism in older adults (Koopman, 2011; Tagawa et al., 2020), contributing to the synergistic effects of training on increase in muscle mass and improvement in physical function (Coelho-Júnior et al., 2022; Tagawa et al., 2022). Serum albumin concentration is a common biomarker used to assess the nutritional status of proteins. A low albumin concentration was reported to be associated with slower gait speed and malnutrition (Kobayashi et al., 2023), indicating that it may impair the effects of resistance training (Sawada et al., 2021). However, in another study, the albumin concentration showed no correlation with gait speed or protein intake (Ashikawa et al., 2020; Motokawa et al., 2024). These differences are probably due to the characteristics of the study population. Albumin concentrations decrease in older adults with severe malnutrition (Gatta et al., 2012). However, this may indicate that functional decline is undetectable in relatively healthy middle-aged and older adults. Therefore, it is now considered a predictor of morbidity and mortality in various diseases, rather than an indicator of protein nutritional status (Gatta et al., 2012). The classical blood protein nutritional parameters, including albumin levels, may not necessarily change with improvements in physical function or muscle mass, and the metabolic changes that accompany these improvements have not been fully explained.

Albumin is broadly classified into reduced and oxidized forms depending on its redox state (Anraku et al., 2013). The fraction of mercaptoalbumin in the total albumin, represented as f(HMA), typically accounts for approximately 70%–80% (Oettl and Marsche, 2010). f(HMA) may be a more sensitive protein nutritional parameter than conventional protein nutrition markers, such as albumin, total protein (TP), free amino acids (FAAs), blood urea nitrogen (BUN), and transthyretin (Kuwahata et al., 2017; Motokawa et al., 2024; Tabata et al., 2023; Wada et al., 2017a; Wada et al., 2018; Wada et al., 2020; Wada et al., 2021; Shibasaki et al., 2025). This parameter decreases rapidly in response to insufficient protein intake (Kuwahata et al., 2017; Wada et al., 2020; Wada et al., 2021), and is correlated with the fractional synthesis rate of albumin in rats (Wada et al., 2017b). This suggests that a similar mechanism may occur in humans, which requires further validation in human studies. Moreover, potential protein deficiency is reflected only by f(HMA) and not by conventional markers in pregnant women and older adults (Motokawa et al., 2024; Tabata et al., 2023; Shibasaki et al., 2025). Thus, f(HMA) has been proposed as an indicator of the state of protein metabolism in the human body (Tabata et al., 2021). Besides, f(HMA) is related to physical function. It reflects the physical function in older adults (Ashikawa et al., 2020; Ito et al., 2021), and is correlated with peak oxygen consumption in patients with cardiac disease (Ishimaru et al., 2023). Thus, f(HMA) is a protein nutritional parameter associated with physical function and may serve as an objective indicator of the efficacy of common protein nutrition and training interventions for improving physical function in older adults. However, previous reports were cross-sectional studies, and changes in f(HMA) in response to training effects remain unclear.

We hypothesized that f(HMA) increases with the improvement of physical function through resistance training. In this study, we aimed to examine the relationship between f(HMA) and the effects of resistance training in healthy Japanese middle-aged and elderly adults.

## 2 Materials and methods

This paper reports the findings of two studies—Study 1, in which f(HMA) was measured as an exploratory factor in addition to the assessment of general blood biochemistry for all participants in 2022 and 2023, and Study 2, a secondary analysis of Study 1 data, focusing on the relationship between f(HMA) and physical function. Study 1, which was aimed at developing a preventive exercise program for locomotive syndrome, was part of repeated examinations scheduled to be conducted annually between 2022 and 2028 and was approved by the Ethics Committee of Juntendo University (Approval Number: 2022–42; date: 1 January 2021). Study 2 was also approved by the Japan Conference of Clinical Research, Tokyo, Japan (Approval Number: ENS-01; date: 31 May 2024), and opt-outs for data use were implemented. Study 1 was registered in the UMIN-Clinical Trial Registry (CTR, ID: UMIN 000042759; date of registration: 15 December 2021).

### 2.1 Study 1

#### 2.1.1 Participants and study ethics

The study participants were healthy community-dwelling, middle-aged, and older Japanese residents. All individuals were recruited through printed media, such as flyers and posters, distributed or displayed in the community or public facilities, and were informed of the methods, procedures, and risks of the study. A total of 48 individuals (11 males and 37 females) agreed to participate in the study, and written informed consent was obtained from them prior to the study. We excluded individuals who did not follow our instructions, who requested to withdraw from study, or those with medical conditions that could limit their ability to participate in the training program based on the decision of the physician in charge. Five individuals were excluded—two were unable to provide blood samples, two declined to continue for personal reasons, and one was unable to continue for medical reasons. Ultimately, 43 participants (10 males and 33 females) completed the study protocol. This study was conducted in accordance with the principles of the Declaration of Helsinki.

#### 2.1.2 Training program

The participants engaged in a low-load body weight-based resistance training program twice a week for 12 weeks under supervision, as reported in previous studies (Ozaki et al., 2020; Sawada et al., 2021). The program consisted of nine types of exercises: squats, push-ups, crunches, hip lifts, heel raises, split squats, seated rows, shoulder presses, and arm curls. An elastic band (Thera-Band, the Hygenic Corporation, Akron, OH) was used for the last three exercises. During the first 2 weeks, the participants performed three sets of eight repetitions of only four exercises: squats, push-ups, crunches, and hip lifts, with 60 s of rest between each set. In each repetition, they were instructed to spend 3 s in both the concentric and eccentric phases. The participants were instructed to continue the program until they became aware of muscle fatigue. The intensity of the exercise program was gradually increased by progressively increasing the number of exercises and number of repetitions per set, and decreasing the rest intervals between exercises every 2 weeks.

#### 2.1.3 Anthropometric measurement

Physical fitness tests were conducted before and after the training program, and as part of these tests, height, weight, and body composition were assessed. Height was measured using a portable stadiometer (seca213, seca Nihon, Japan). Body weight was measured, and body composition was estimated using a bioelectrical impedance analyzer (InBody 770, InBody Japan Inc., Japan). First, we asked the participants whether they used a cardiac pacemaker; then, we wiped the palms of their hands and soles of their feet with InBody Tissue, measured their height and weight, and assessed their body composition. Participants using cardiac pacemakers were only required to have their height and weight measured, and none of the participants in this study used a cardiac pacemaker.

#### 2.1.4 Physical function

Gait speed is recommended by the Asian Working Group for Sarcopenia (AWGS 2019) for the evaluation of physical function (Chen et al., 2020). Two types of gait speed, usual and maximum, were measured from the time taken to walk a distance of 10 meters. The participants were instructed to walk at their usual and maximum speeds without running. They were given an extra 2 meters at the beginning and end of the 10-meters walking distance for acceleration and deceleration.

#### 2.1.5 Dietary intakes of macronutrients

The dietary intakes of macronutrients, protein, fat, and carbohydrates were estimated using a brief-type self-administered diet history questionnaire (BDHQ). The BDHQ is a validated questionnaire consisting of 58 food items commonly consumed in Japan, which is used to estimate dietary intake in Japan (Kobayashi et al., 2011). Participants were asked to report how frequently they consumed each food in the past month to estimate their individual nutrient intake. Energy adjustment was performed for each nutrient according to a previously described method (Willett et al., 1997). Protein intake was divided into animal and vegetable sources and adjusted for body weight, as indicated by the respective recommended daily allowances (RDA) (Ministry of Health, Labour and Welfare, 2020).

#### 2.1.6 Blood biochemistry

Blood sample was drawn from the antecubital vein after at least 2 h of fasting, and the obtained serum sample was stored at −80 °C until analysis. Serum f(HMA) was determined using high-performance liquid chromatography (HPLC) as previously described (Tabata et al., 2023). Two microliter of serum was diluted 60 times with phosphate-buffered saline (pH 7.4), and 20 µL of the diluted sample was subjected to HPLC. Serum total protein (TP) was analyzed using the Biuret method and albumin concentrations were analyzed using the modified bromocresol purple method (Muramoto et al., 1999; Smith et al., 1985). FAA was quantitated using an L-8900 amino acid analyzer (Hitachi High-Technologies, Tokyo, Japan), and the concentrations of essential FAAs (EFAAs) and branched-chain amino acids (BCAAs) were calculated (Sakata et al., 2022). For the quantitation of FAAs, serum was diluted fourfold with 6.66% trichloroacetic acid and centrifuged at 15,000 × g for 15 min at 4 °C to precipitate the proteins. The supernatant was passed through a 0.22 µm filter and analyzed. Glucose was analyzed using the UV method with hexokinase (Lutz and Flückiger, 1975), insulin was analyzed using a chemiluminescent enzyme immunoassay (Shen et al., 2019), triglycerides were analyzed using the GPO-POD method, total cholesterol was analyzed using the CHOD-POD method, and HDL- and LDL-cholesterol were analyzed using the direct measurement method (Bucolo and David, 1973).

#### 2.1.7 Statistical analyses of characteristics of participants

Values are presented as mean ± standard deviation (SD). Paired *t*-tests were used for comparisons before and after the intervention. All statistical analyses were performed using R (version 4.3.1; R Foundation for Statistical Computing), and statistical significance was set at *P*-value < 0.05.

### 2.2 Study 2—Regression analyses

Multivariable linear regression analyses were performed to assess the relationships between serum nutritional parameters and gait speed before intervention and between the changing rate of serum nutritional parameters and the changing rate of gait speed after the intervention. Given the relatively small sample size, only age and sex were included as covariates in the regression models to minimize the risk of overfitting while adjusting for potential confounders. Age and sex were selected because they are both basic confounding factors and are associated with physical function and biomarkers (Ito et al., 2021). The normality of the residuals was checked using the Shapiro–Wilk test, and the dependent variables were log-transformed when normality was not rejected. Multicollinearity was assessed using variance inflation factors (VIFs). All statistical analyses were performed using R (version 4.3.1; R Foundation for Statistical Computing), and statistical significance was set at *P*-value < 0.05.

## 3 Results

### 3.1 Study 1—Characteristics and changes in parameters before and after the intervention

The average age of participants was 67.3 ± 8.0 years. In terms of anthropometric data before and after the intervention, the BMI values were 23.1 ± 3.2 and 23.3 ± 3.1 kg/m^2^, respectively. Among the serum nutritional parameters, f(HMA) and HDL-cholesterol levels significantly increased (*P* = 0.006 and 0.041, respectively; Table 1), whereas albumin levels significantly decreased after the intervention (*P* < 0.001; Table 1). Other parameters, including physical function and dietary intake of macronutrients, did not change significantly (Table 1).

**TABLE 1:** Characteristics of participants.

Characteristics (*n* = 43)	Pre	Post	Mean differences (post–pre)	*P*-value^a^
Physical function				
Usual gait speed, m/s	1.44 ± 0.17	1.41 ± 0.16	−0.03 ± 0.14	0.241
Maximal gait speed, m/s	1.90 ± 0.21	1.95 ± 0.24	0.71 ± 1.35	0.079
Blood biochemistry				
TP, g/dL	7.5 ± 0.5	7.4 ± 0.4	−0.03 ± 0.28	0.548
Albumin, g/dL	4.5 ± 0.2	4.3 ± 0.2	−0.15 ± 0.18	<0.001***
f(HMA), %	71.29 ± 3.81	72.84 ± 3.58	1.55 ± 3.49	0.006**
EFAAs, mM	1.0 ± 0.3	1.1 ± 0.2	0.04 ± 0.24	0.306
BCAAs, mM	0.5 ± 0.2	0.5 ± 0.1	0.01 ± 0.14	0.472
Glucose, mg/dL	107.6 ± 22.1	114.0 ± 32.5	6.4 ± 33.4	0.216
Insulin, µU/mL	21.8 ± 42.8	20.7 ± 18.6	−1.06 ± 39.3	0.861
Triglyceride, mg/dL	142.2 ± 85.9	141.7 ± 80.6	−0.53 ± 66.9	0.958
Total cholesterol, mg/dL	217.0 ± 46.7	220.1 ± 44.3	3.07 ± 19.9	0.318
HDL-cholesterol, mg/dL	64.3 ± 16.9	67.3 ± 16.7	2.95 ± 7.52	0.041*
LDL-cholesterol, mg/dL	126.4 ± 36.9	126.5 ± 36.5	0.05 ± 15.7	0.985
Macronutrient intakes^b^				
Energy intake, kcal/d	1847 ± 564	1987 ± 567	140.2 ± 504	0.075
Total protein, g/kg/d	1.54 ± 0.43	1.51 ± 0.48	−0.21 ± 0.39	0.427
Animal protein, g/kg/d	0.98 ± 0.39	0.94 ± 0.45	−0.05 ± 0.31	0.315
Plant protein, g/kg/d	0.56 ± 0.16	0.57 ± 0.13	1.14 ± 4.90	0.134
Carbohydrate, g/d	225 ± 46.4	232 ± 48.0	7.14 ± 39.5	0.243
Fat, g/d	65.6 ± 12.6	63.0 ± 12.6	−2.59 ± 13.6	0.217

Data are presented as the mean ± standard deviation.

^a^ Differences were considered statistically significant at *p < 0.05, **p < 0.01, and ***p < 0.001.

^b^ Energy adjustment was performed for each protein intake using the residual method.

Abbreviations: f(HMA), fraction of human mercaptoalbumin in total serum albumin; TP, total protein; EFAAs, essential free amino acids; BCAAs, branched-chain amino acids; HDL-cholesterol: high-density lipoprotein-cholesterol; LDL-cholesterol, low-density lipoprotein-cholesterol.

### 3.2 Study 2

#### 3.2.1 Multivariable linear regression analysis of serum nutritional parameters and gait speed before the intervention

f(HMA) showed significant positive correlation with both usual gait speed (*β* = 0.326, *P* = 0.045) and maximum gait speed (*β* = 0.331, *P* = 0.036, Table 2). LDL-cholesterol levels showed a significant negative correlation with both usual (*β* = −0.428, *P* = 0.009) and maximum (*β* = 0.330, *P* = 0.043, Supplementary Table S1) gait speeds. No correlation was noted between other nutritional parameters (albumin, TP, EFAAs, BCAAs, glucose, insulin, triglyceride, total and HDL-cholesterol) and either of the two types of gait speeds (*β* = −0.319 to 0.278, *P* = 0.065–0.999, respectively, Table 2; Supplementary Table S1).

**TABLE 2:** Multivariable linear regression analysis of serum protein nutritional biomarkers vs. gait speed before training.

Independent value	Dependent value^a^ ^,^ ^b^ (pre-gait speed [m/s])			
	Usual^c^		Maximal	
	*β* (95% CI)	*P*-value^a^	*β* (95% CI)	*P*-value^a^
Protein metabolites				
f(HMA), %	0.326 (0.007 to 0.645)	0.045*	0.331 (0.023 to 0.639)	0.036*
Albumin, g/dL	0.079 (−0.261 to 0.419)	0.640	0.000 (−0.329 to 0.330)	0.999
TP, g/dL	−0.058 (−0.400 to 0.284)	0.732	−0.090 (−0.421 to 0.241)	0.587
EFAAs, mM	0.170 (−0.183 to 0.523)	0.337	0.099 (−0.246 to 0.444)	0.566
BCAAs, mM	0.073 (−0.295 to 0.441)	0.690	0.061 (−0.296 to 0.419)	0.730

^a^ Differences were considered statistically significant at *p < 0.05, **p < 0.01, and ***p < 0.001.

^b^ Adjusted for age and sex.

^c^ Log-transformed in analysis with EFAAs.

Abbreviations: CI, confidence interval; f(HMA), fraction of human mercaptoalbumin in total serum albumin; TP, total protein; EFAAs, essential free amino acids; BCAAs, branched-chain amino acids.

#### 3.2.2 Multivariable linear regression analysis of the changing rate of serum nutritional parameters and gait speed

The changing rate of f(HMA) showed a significant positive correlation with the changing rate of maximum gait speed (*β* = 0.456, *P* = 0.004, Table 3), but not with the usual gait speed (*β* = 0176, *P* = 0.282, Table 3). No correlation was noted between other nutritional parameters (albumin, TP, EFAAs, BCAAs, glucose, insulin, triglyceride, and cholesterol) and gait speed (*β* = −0.220 to 0.290, *P* = 0.068 to 0.969, Table 3; Supplementary Table S2).

**TABLE 3:** Multivariable linear regression analysis of improvement rate of serum protein nutritional biomarkers vs. gait speed.

Independent value	Dependent value^a^ ^,^ ^b^ (improvement of gait speed [%])			
	Usual		Maximal	
	*β* (95% CI)	*P*-value^a^	*β* (95% CI)	*P*-value^a^
Protein metabolite				
f(HMA), %	0.176 (−0.151 to 0.503)	0.282	0.456 (0.153 to 0.286)	0.004**
Albumin, %	0.092 (−0.230 to 0.414)	0.565	0.006 (−0.325 to 0.338)	0.969
TP, %	0.111 (−0.214–0.437)	0.493	−0.220 (−0.546 to 0.106)	0.181
EFAAs, %	−0.171 (−0.493 to 0.150)	0.287	−0.002 (−0.334 to 0.330)	0.991
BCAAs, %	−0.190 (−0.517 to 0.136)	0.220	−0.077 (−0.409 to 0.255)	0.462

^a^ Differences were considered statistically significant at *p < 0.05, **p < 0.01, and ***p < 0.001.

^b^ Adjusted for age and sex.

Abbreviations: CI, confidence interval; f(HMA), fraction of human mercaptoalbumin in total serum albumin; TP, total protein; EFAAs, essential free amino acids; BCAAs, branched-chain amino acids.

## 4 Discussion

The present study demonstrates that f(HMA) is associated with both usual and maximal gait speeds in healthy middle-aged and older adults. Moreover, it changed with the changes in the maximal gait speeds through training. These results indicate that a change in f(HMA) is associated with training effectiveness in physical function. f(HMA) can potentially be useful in early detection of physical decline in middle-aged and older adults. It can also be used to predict the effectiveness of training programs aimed at improving physical function during rehabilitation.

Previous studies have reported that f(HMA) is a more sensitive indicator of physical function than albumin concentration (Ashikawa et al., 2020; Ito et al., 2021). A similar relationship was observed in the present study before the intervention (*β* = 0.326–0.331, *P* = 0.045 and 0.036, respectively, Table 2). This indicates that a qualitative change occurs prior to a decrease in total concentration, as evident from the ratio of mercaptoalbumin to non-mercaptoalbumin (Ashikawa et al., 2020; Tabata et al., 2021). Therefore, f(HMA) can used to predict the decline in physical function. Notably, the association between gait speed and f(HMA) was demonstrated even in a healthy population that did not meet the gait speed criteria as per AWGS 2019 (Chen et al., 2020). This suggests that a decline in gait speed may have already begun in some individuals, even when they did not meet the criteria.

Resistance training is widely accepted to stimulate muscle protein synthesis (MPS) (Kramer et al., 2017), thereby improving muscle mass and physical function (Ozaki et al., 2020). However, in middle-aged and older adults, these effects can vary owing to anabolic resistance (Koopman, 2011; Kumar et al., 2009). Therefore, individuals with enhanced protein metabolic turnover may derive greater benefits from training. In animal studies, f(HMA) is considered an indicator of the state of protein metabolic turnover (Tabata et al., 2021; Wada et al., 2018) as it correlates with the rate of albumin synthesis in the liver (Wada A. et al., 2023; Wada et al., 2017b). The correlation between f(HMA) and physical function may reflect increased protein metabolism and anabolic resistance in the body.

Resistance training enhances physical function and potentially increases f(HMA). Indeed, f(HMA) was the only indicator that exhibited changes with the training effects on maximal gait speed (*β* = 0.456, *P* = 0.004, Table 3). Average protein intake was >1.5 g/kg body weight, which exceeded the RDA and did not change before or after the intervention (Table 1). Nevertheless, there were individual differences in training effects. These differences may be due to variations in protein metabolic turnover. Although only pre- and post-intervention status was examined in this study, f(HMA) may change more quickly than does the physical function. Improvement in physical function requires an increase in lower limb muscle mass and function, and the activation of protein metabolism is necessary before such improvement can occur. Although f(HMA) may be influenced by both nutritional status and resistance training, we did not directly investigate the effect of variations in dietary intake. Further studies are warranted to clarify the roles of both dietary factors and exercise in affecting f(HMA) and improvements in physical function.

f(HMA) also decreased with aging and oxidative stress (Anraku et al., 2013; Era et al., 1995; Oettl and Marsche, 2010). The results of the present study contrast with those of previous studies wherein training intervention induced oxidative stress and decreased f(HMA) (Imai et al., 2005; James et al., 2024; Lamprecht et al., 2008). The participants in the previous studies were athletes and the increase in oxidative stress caused by high-intensity training may have been more predominant than the increase in protein metabolism. Therefore, differences in f(HMA) after exercise training may be due to differences in exercise intensity and subject characteristics. However, in the present study, the influence of oxidative stress markers was unclear and requires further validation.

The reason why parameters other than f(HMA) were not associated with physical function is unclear (Tables 2, 3). Albumin is the most abundant protein in the blood and is responsible for maintaining osmotic pressure and transporting hydrophobic molecules (Margarson and Soni, 1998). Albumin has a long half-life that allows it to maintain stable concentrations (Beeken et al., 1962; Gatta et al., 2012). In contrast, f(HMA) was a more sensitive marker of protein nutritional status in older adults than albumin (Motokawa et al., 2024). In early stages of decline in the synthetic capacity, the production of reduced albumin decreases, whereas the half-life of oxidized albumin is prolonged (Wada et al., 2017b). This mechanism helps in maintaining the total albumin concentration above the clinical threshold of 3.5 g/dL, thereby masking early impairments in protein metabolism when using total albumin concentration as a marker. Similarly, TTR, TP, and amino acids present in the blood maintain homeostasis and may be less sensitive indicators than f(HMA). In the present study, exercise intervention led to an increase in f(HMA) and a decrease in the fraction of non-mercaptoalbumin (*P* = 0.006; Table 1). In addition, albumin levels were decreased (*P* < 0.001, Table 1). Although the cause of the decrease in albumin levels was unclear, the values did not fall below 3.5 g/dL. Resistance training may have facilitated protein synthesis, increasing the production of mercaptoalbumin, whereas the concomitant and even greater decrease in non-mercaptoalbumin could explain the overall reduction in total albumin concentration. This could potentially explain the lack of an association between albumin levels and changes in gait speed.

This study had several limitations. First, the independent effects of exercise training on f(HMA) remain unclear. The relationship between f(HMA) and exercise has only been investigated in a few studies. Therefore, it is necessary to study the relationship between changes in physical function and f(HMA) as a training effect in greater detail. Second, we did not measure specific markers of oxidative stress, which could have helped in further distinguishing nutritional effects and redox-related changes. Third, the sample size in this study was small. The participants were health-conscious individuals who actively engaged in the training program, with a predominance of female participants. Sex and age can influence f(HMA) and physical performance (Ito et al., 2021). Although age and sex were included as covariates to adjust for confounding factors, subgroup analyses could not be performed due to the limited sample size. Thus, future studies are warranted to validate our findings using larger and more diverse samples, including populations with frailty and sarcopenia, and to employ subgroup analyses to further explore these effects. Fourth, this study was not a randomized controlled trial and did not include a control group; therefore, potential biases could not be excluded. The inclusion of a control group in future studies would allow for a more rigorous evaluation of f(HMA) efficacy.

In conclusion, the present study demonstrates that serum f(HMA) before resistance training is associated with physical function in healthy middle-aged and older adults. This study provides the first evidence that f(HMA) changes with physical function during resistance training. This evidence could help predict the efficacy of training programs aimed at improving physical function in middle-aged and older adults undergoing rehabilitation or preventive interventions aimed at reducing the functional decline.

## Data Availability

The original contributions presented in the study are included in the article/Supplementary Material, further inquiries can be directed to the corresponding authors.

## References

[B1] Abe T. Kitamura A. Taniguchi Y. Amano H. Seino S. Yokoyama Y. (2019). Pathway from gait speed to incidence of disability and mortality in older adults: a mediating role of physical activity. Maturitas 123, 32–36. 10.1016/j.maturitas.2019.02.002 31027674

[B2] Anraku M. Chuang V. T. G. Maruyama T. Otagiri M. (2013). Redox properties of serum albumin. Biochim. Biophys. Acta 1830, 5465–5472. 10.1016/j.bbagen.2013.04.036 23644037

[B3] Ashikawa H. Adachi T. Ueyama J. Yamada S. (2020). Association between redox state of human serum albumin and exercise capacity in older women: a cross-sectional study. Geriatr. Gerontol. Int. 20, 256–260. 10.1111/ggi.13849 31854142

[B4] Beeken W. L. Volwiler W. Goldsworthy P. D. Garby L. E. Reynolds W. E. Stogsdill R. (1962). Studies of I-131-albumin catabolism and distribution in normal young male adults. J. Clin. Invest. 41, 1312–1333. 10.1172/JCI104594 13866511 PMC291046

[B5] Bucolo G. David H. (1973). Quantitative determination of serum triglycerides by the use of enzymes. Clin. Chem. 19, 476–482. 10.1093/clinchem/19.5.476 4703655

[B6] Chen L. K. Woo J. Assantachai P. Auyeung T.-W. Chou M.-Y. Iijima K. (2020). Asian Working Group for Sarcopenia: 2019 consensus update on sarcopenia diagnosis and treatment. J. Am. Med. Dir. Assoc. 21, 300–307. 10.1016/j.jamda.2019.12.012 32033882

[B7] Coelho-Júnior H. J. Calvani R. Tosato M. Landi F. Picca A. Marzetti E. (2022). Protein intake and physical function in older adults: a systematic review and meta-analysis. Ageing Res. Rev. 81, 101731. 10.1016/j.arr.2022.101731 36087703

[B8] Colón-Emeric C. S. McDermott C. L. Lee D. S. Berry S. D. (2024). Risk assessment and prevention of falls in older community-dwelling adults. JAMA 331, 1397–1406. 10.1001/jama.2024.1416 38536167 PMC12224174

[B9] Deutz N. E. P. Bauer J. M. Barazzoni R. Biolo G. Boirie Y. Bosy-Westphal A. (2014). Protein intake and exercise for optimal muscle function with aging: recommendations from the ESPEN Expert Group. Clin. Nutr. 33, 929–936. 10.1016/j.clnu.2014.04.007 24814383 PMC4208946

[B10] Era S. Kuwata K. Imai H. Nakamura K. Hayashi T. Sogami M. (1995). Age-related change in redox state of human serum albumin. Biochim. Biophys. Acta 1247, 12–16. 10.1016/0167-4838(94)00166-e 7873580

[B11] Gatta A. Verardo A. Bolognesi M. (2012). Hypoalbuminemia. Intern emerg. Med. 7, 193–199. 10.1007/s11739-012-0802-0 23073857

[B12] Imai H. Era S. Hayashi T. Negawa T. Matsuyama Y. Okihara K. (2005). Effect of propolis supplementation on the redox state of human serum albumin during high-intensity Kendo training. Adv. Exerc. Sports Physiol. 11, 109–113. Available online at: https://mol.medicalonline.jp/archive/search?jo=dt4adexp&ye=2005&vo=11&issue=3 .

[B13] Ishimaru Y. Adachi T. Ashikawa H. Hori M. Shimozato T. Ohtake H. (2023). Association between the redox state of human serum albumin and exercise capacity in patients with cardiac disease. Am. J. Cardiol. 189, 56–60. 10.1016/j.amjcard.2022.11.034 36508763

[B14] Ito S. Nakashima H. Ando K. Kobayashi K. Machino M. Seki T. (2021). Human nonmercaptalbumin is a new biomarker of motor function. J. Clin. Med. 10, 2464. 10.3390/jcm10112464 34199414 PMC8199584

[B15] James C. Dugan C. W. Boyd C. Fournier P. A. Arthur P. G. (2024). Temporal tracking of cysteine 34 oxidation of plasma albumin as a biomarker of muscle damage following a bout of eccentric exercise. Eur. J. Appl. Physiol. 124, 2639–2650. 10.1007/s00421-024-05488-1 38627299 PMC11365830

[B16] Kobayashi S. Murakami K. Sasaki S. Okubo H. Hirota N. Notsu A. (2011). Comparison of relative validity of food group intakes estimated by comprehensive and brief-type self-administered diet history questionnaires against 16 d dietary records in Japanese adults. Public Health Nutr. 14, 1200–1211. 10.1017/S1368980011000504 21477414

[B17] Kobayashi K. Nishida T. Sakakibara H. (2023). Factors associated with low albumin in community-dwelling older adults aged 75 years and above. Int. J. Environ. Res. Public Health 20, 6994. 10.3390/ijerph20216994 37947552 PMC10648125

[B18] Koopman R. (2011). Dietary protein and exercise training in ageing. Proc. Nutr. Soc. 70, 104–113. 10.1017/S0029665110003927 21092364

[B19] Kramer I. F. Verdijk L. B. Hamer H. M. Verlaan S. Luiking Y. C. Kouw I. W. K. (2017). Both basal and post-prandial muscle protein synthesis rates, following the ingestion of a leucine-enriched whey protein supplement, are not impaired in sarcopenic older males. Clin. Nutr. 36, 1440–1449. 10.1016/j.clnu.2016.09.023 27743615

[B20] Kumar V. Atherton P. Smith K. Rennie M. J. (2009). Human muscle protein synthesis and breakdown during and after exercise. J. Appl. Physiol. 106, 2026–2039. 10.1152/japplphysiol.91481.2008 19164770

[B21] Kuwahata M. Hasegawa M. Kobayashi Y. Wada Y. Kido Y. (2017). An oxidized/reduced state of plasma albumin reflects malnutrition due to an insufficient diet in rats. J. Clin. Biochem. Nutr. 60, 70–75. 10.3164/jcbn.16-33 28163385 PMC5281528

[B22] Lamprecht M. Greilberger J. F. Schwaberger G. Hofmann P. Oettl K. (2008). Single bouts of exercise affect albumin redox state and carbonyl groups on plasma protein of trained men in a workload-dependent manner. J. Appl. Physiol. 104, 1611–1617. 10.1152/japplphysiol.01325.2007 18420715

[B23] Lutz R. A. Flückiger J. (1975). Kinetic determination of glucose with the GEMSAEC (ENI) centrifugal analyzer by the glucose dehydrogenase reaction, and comparison with two commonly used procedures. Clin. Chem. 21, 1372–1377. 10.1093/clinchem/21.10.1372 1157301

[B24] Margarson M. P. Soni N. (1998). Serum albumin: touchstone or totem?. Anaesthesia 53, 789–803. 10.1046/j.1365-2044.1998.00438.x 9797524

[B25] Mende E. Moeinnia N. Schaller N. Weiß M. Haller B. Halle M. (2022)). Progressive machine-based resistance training for prevention and treatment of sarcopenia in the oldest old: a systematic review and meta-analysis. Exp. Gerontol. 163, 111767. 10.1016/j.exger.2022.111767 35318104

[B26] Ministry of Health, Labour and Welfare (2020). Dietary reference intakes for Japanese. Available online at: https://www.mhlw.go.jp/content/001151422.pdf (Accessed June 16, 2020).

[B27] Motokawa K. Shirobe M. Iwasaki M. Wada Y. Tabata F. Shigemoto K. (2024). Serum albumin redox state as an indicator of dietary protein intake among community-dwelling older adults. Clin. Nutr. ESPEN 63, 157–161. 10.1016/j.clnesp.2024.06.028 38944830

[B28] Muramoto Y. Matsushita M. Irino T. (1999). Reduction of reaction differences between human mercaptalbumin and human nonmercaptalbumin measured by the bromocresol purple method. Clin. Chim. Acta 289, 69–78. 10.1016/S0009-8981(99)00158-8 10556654

[B29] Oettl K. Marsche G. (2010). Redox state of human serum albumin in terms of cysteine-34 in health and disease. Methods Enzymol. 474, 181–195. 10.1016/S0076-6879(10)74011-8 20609911

[B30] Ozaki H. Sawada S. Osawa T. Natsume T. Yoshihara T. Deng P. (2020). Muscle size and strength of the lower body in supervised and in combined supervised and unsupervised low-load resistance training. J. Sport Sci. Med. 19, 721–726. Available online at: https://www.jssm.org/jssm-19-721.xml-Fulltext . 33239946 PMC7675625

[B31] Peng T. C. Chen W. L. Wu L. W. Chang Y. W. Kao T. W. (2020). Sarcopenia and cognitive impairment: a systematic review and meta-analysis. Clin. Nutr. 39, 2695–2701. 10.1016/j.clnu.2019.12.014 31917049

[B32] Perera S. Patel K. V. Rosano C. Rubin S. M. Satterfield S. Harris T. (2016). Gait speed predicts incident disability: a pooled analysis. J. Gerontol. A Biol. Sci. Med. Sci. 71, 63–71. 10.1093/gerona/glv126 26297942 PMC4715231

[B33] Sakata Y. Yago T. Mori S. Seto N. Matsunaga Y. Nakamura H. (2022). Time courses of gastric volume and content after different types of casein ingestion in healthy men: a randomized crossover study. J. Nutr. 152, 2367–2375. 10.1093/jn/nxac158 36774103

[B34] Sawada S. Ozaki H. Natsume T. Nakano D. Deng P. Yoshihara T. (2021). Serum albumin levels as a predictive biomarker for low-load resistance training programs’ effects on muscle thickness in the community-dwelling elderly Japanese population: interventional study result. BMC Geriatr. 21, 464. 10.1186/s12877-021-02403-7 34407763 PMC8371758

[B35] Shen Y. Prinyawiwatkul W. Xu Z. (2019). Insulin: a review of analytical methods. Analyst 144, 4139–4148. 10.1039/C9AN00112C 31143899

[B51] Shibasaki T. Nakamura H. Kamimura T. Tabata F. Kawakami S. Inubashiri M. (2025). Relationship between dietary protein intake and serum essential free amino acid concentrations in Japanese pregnant women: an observational study. BMC Pregnancy Childbirth. 25, 852. 10.1186/s12884-025-07962-w 40814083 PMC12351771

[B36] Smith P. K. Krohn R. I. Hermanson G. T. Mallia A. K. Gartner F. H. Provenzano M. D. (1985). Measurement of protein using bicinchoninic acid. Anal. Biochem. 150, 76–85. 10.1016/0003-2697(85)90442-7 3843705

[B37] Tabata F. Wada Y. Kawakami S. Miyaji K. (2021). Serum albumin redox states: more than oxidative stress biomarker. Antioxidants (Basel) 10, 503. 10.3390/antiox10040503 33804859 PMC8063786

[B48] Tabata F. Wada Y. Shibasaki T. Kawakami S. Inubashiri M. Hosaka M. (2023). A lower ratio of reduced to total albumin in serum is associated with protein nutritional status of pregnant women in Japan. Nutr. Res. 114, 1–12. 10.1016/j.nutres.2023.03.006 37079948

[B38] Tagawa R. Watanabe D. Ito K. Ueda K. Nakayama K. Sanbongi C. (2020). Dose-response relationship between protein intake and muscle mass increase: a systematic review and meta-analysis of randomized controlled trials. Nutr. Rev. 79, 66–75. 10.1093/nutrit/nuaa104 33300582 PMC7727026

[B39] Tagawa R. Watanabe D. Ito K. Otsuyama T. Nakayama K. Sanbongi C. (2022). Synergistic effect of increased total protein intake and strength training on muscle strength: a dose-response meta-analysis of randomized controlled trials. Sport Med. Open 8, 110. 10.1186/s40798-022-00508-w 36057893 PMC9441410

[B40] Taniguchi Y. Kitamura A. Seino S. Murayama H. Amano H. Nofuji Y. (2017). Gait performance trajectories and incident disabling dementia among community-dwelling older Japanese. J. Am. Med. Dir. Assoc. 18, 192.e13–192. 10.1016/j.jamda.2016.10.015 28049615

[B41] Toots A. Rosendahl E. Lundin-Olsson L. Nordström P. Gustafson Y. Littbrand H. (2013). Usual gait speed independently predicts mortality in very old people. A population-based study. J. Am. Med. Dir. Assoc. 14, 529.e1–529.e6. 10.1016/j.jamda.2013.04.006 23706405

[B42] Wada A. Nakamura M. Kobayashi K. Kuroda A. Harada D. Kido S. (2023). Effects of amino acids and albumin administration on albumin metabolism in surgically stressed rats: a basic nutritional study. J. Parenter. Enter. Nutr. 47, 399–407. 10.1002/jpen.2472 36597725

[B43] Wada Y. Takeda Y. Kuwahata M. (2017a). Potential role of amino acid/protein nutrition and exercise in serum albumin redox state. Nutrients 10, 17. 10.3390/nu10010017 29295548 PMC5793245

[B44] Wada Y. Sato Y. Miyazaki K. Takeda Y. Kuwahata M. (2017b). The reduced/oxidized state of plasma albumin is modulated by dietary protein intake partly via albumin synthesis rate in rats. Nutr. Res. 37, 46–57. 10.1016/j.nutres.2016.12.003 28215314

[B45] Wada Y. Komatsu Y. Izumi H. Shimizu T. Takeda Y. Kuwahata M. (2018). Increased ratio of non-mercaptalbumin-1 among total plasma albumin demonstrates potential protein undernutrition in adult rats. Front. Nutr. 5, 64. 10.3389/fnut.2018.00064 30090810 PMC6068262

[B46] Wada Y. Izumi H. Shimizu T. Takeda Y. (2020). A more oxidized plasma albumin redox state and lower plasma HDL particle number reflect low-protein diet ingestion in adult rats. J. Nutr. 150, 256–266. 10.1093/jn/nxz223 31552421

[B47] Wada Y. Ehara T. Tabata F. Komatsu Y. Izumi H. Kawakami S. (2021). Maternal serum albumin redox state is associated with infant birth weight in Japanese pregnant women. Nutrients 13, 1764. 10.3390/nu13061764 34067270 PMC8224550

[B49] Willett W. C. Howe R. Kushi L. H. (1997). Adjustment for total energy intake in epidemiologic studies. Am. J. Clin. Nutr. 65, 1220S–1231S. 10.1093/ajcn/65.4.1220S 9094926

[B50] Yoshimura Y. Wakabayashi H. Yamada M. Kim H. Harada A. Arai H. (2017). Interventions for treating sarcopenia: a systematic review and meta-analysis of randomized controlled studies. J. Am. Med. Dir. Assoc. 18, 553.e1–553. 10.1016/j.jamda.2017.03.019 28549707

