# Supplementary material for: The effect of resistance training on physical function is associated with changes in serum albumin redox state in middle-aged and older Japanese adults: a Quasi-experimental study
Source: Front Physiol. 2025 Sep 17;16:1649300. doi: 10.3389/fphys.2025.1649300 (PMC12485620; doi:10.3389/fphys.2025.1649300)
Supplement: Supplementary file 1 [file DataSheet1.pdf]

Supplementary Table S1. Multivariable linear regression analysis of serum carbohydrate and lipid nutritional biomarkers *vs.* gait speed before training.

Independent value	Dependent value[1,2] (pre-gait speed [m/s])			
	Usual[3]		Maximal[3]	
	β (95% CI)	*P*-value[1]	β (95% CI)	*P*-value[1]
Carbohydrate metabolites				
Glucose, mg/dL	0.278 (−0.036 to 0.592)	0.081	−0.088 (−0.403 to 0.228)	0.577
Insulin, µU/mL	0.157 (−0.163 to 0.477)	0.327	−0.213 (−0.519 to 0.094)	0.168
Lipid metabolites				
Triglyceride, mg/dL	−0.013 (−0.358 to 0.332)	0.939	0.008 (−0.327 to 0.343)	0.963
Total cholesterol, mg/dL	−0.319 (−0.659 to 0.021)	0.065	−0.273 (−0.605 to 0.060)	0.105
HDL-cholesterol, mg/dL	0.057 (−0.277 to 0.331)	0.733	0.057 (−0.266 to 0.380)	0.723
LDL-cholesterol, mg/dL	−0.428 (−0.744 to −0.112)	0.009**	−0.330 (−0.649 to −0.011)	0.043*

[1] Differences were considered statistically significant at * $p < 0.05$, ** $p < 0.01$, and *** $p < 0.001$.

[2] Adjusted for age and sex.

[3] Log-transformed in analysis with LDL-cholesterol.

Abbreviations: CI, confidence interval; HDL-cholesterol, high-density lipoprotein; LDL-cholesterol, low-density lipoprotein.

Supplementary Table S2. Multivariable linear regression analysis of improvement rate of serum carbohydrate and lipid nutritional biomarkers *vs.* gait speed.

Independent value	Dependent value[1] (improvement of gait speed [%])			
	Usual		Maximal	
	β (95% CI)	*P*-value	*β* (95% CI)	*P*-value
Carbohydrate metabolite				
Glucose, %	0.222 (−0.098 to 0.541)	0.168	0.233 (−0.092 to 0.558)	0.154
Insulin, %	0.213 (−0.100 to 0.526)	0.177	0.290 (−0.023 to 0.603)	0.068
Lipid metabolite				
Triglyceride, %	−0.098 (−0.417 to 0.221)	0.538	0.067 (−0.258 to 0.393)	0.678
Total cholesterol, %	0.199 (−0.127 to 0.525)	0.224	0.065 (−0.272 to 0.403)	0.698
HDL-cholesterol, %	0.182 (−0.157 to 0.522)	0.284	−0.158 (−0.507 to 0.191)	0.365
LDL-cholesterol, %	0.186 (−0.143 to 0.514)	0.259	0.069 (−0.27 to 0.408)	0.683

[1] Adjusted for age and sex.

Abbreviations: CI, confidence interval; HDL-cholesterol, high-density lipoprotein; LDL-cholesterol, low-density lipoprotein.
